# Prospective Design, Rapid Prototyping, and Testing of Smart Dressings, Drug Delivery Patches, and Replacement Body Parts Using Microscopy Aided Design and ManufacturE (MADAME)

**DOI:** 10.3389/fmed.2018.00348

**Published:** 2018-12-13

**Authors:** Hans Jörg Sidler, Jacob Duvenage, Eric J. Anderson, Joanna Ng, Daniel J. Hageman, Melissa L. Knothe Tate

**Affiliations:** ^1^Institute of Biomedical Engineering and Medical Informatics, Swiss Federal Institute of Technology, Zurich, Switzerland; ^2^MechBio Team, Graduate School of Biomedical Engineering, University of New South Wales, Sydney, NSW, Australia; ^3^Departments of Mechanical & Aerospace Engineering and Biomedical Engineering, Case Western Reserve University, Cleveland, OH, United States; ^4^National Oceanic and Atmospheric Administration, Great Lakes Environmental Research Laboratory, Ann Arbor, MI, United States

**Keywords:** microscopy-aided design and manufacture, advanced materials, imaging, computational modeling, medical devices, smart materials and systems, translational medicine

## Abstract

Natural materials exhibit smart properties including gradients in biophysical properties that engender higher order functions, as well as stimuli-responsive properties which integrate sensor and/or actuator capacities. Elucidation of mechanisms underpinning such smart material properties (i), and translation of that understanding (ii), represent two of the biggest challenges in emulating natural design paradigms for design and manufacture of disruptive materials, parts, and products. Microscopy Aided Design And ManufacturE (MADAME) stands for a computer-aided additive manufacturing platform that incorporates multidimensional (multi-D) printing and computer-controlled weaving. MADAME enables the creation of composite design motifs emulating e.g., patterns of woven protein fibers as well as gradients in different caliber porosities, mechanical, and molecular properties, found in natural tissues, from the skin on bones (periosteum) to tree bark. Insodoing, MADAME provides a means to manufacture a new genre of smart materials, products and replacement body parts that exhibit advantageous properties both under the influence of as well as harnessing dynamic mechanical loads to activate material properties (mechanoactive properties). This Technical Report introduces the MADAME technology platform and its associated machine-based workflow (pipeline), provides basic technical background of the novel technology and its applications, and discusses advantages and disadvantages of the approach in context of current 3 and 4D printing platforms.

## Introduction

With the increasing mean age of the population and pressures on the health care system to increase accessibility to while also decreasing cost of care, there is an acute imperative to develop smarter materials enabling the creation and manufacture of products, devices, replacement tissue body parts, and associated therapeutic approaches. Such products, devices, and approaches will obviate the need for allo- and xeno-grafts (i.e., tissue graft from same species but not the same subject, and respectively from a different species) and their inherent limitations. At the same time, as the regulatory processes regarding combination products and devices become more streamlined, there is a great opportunity to use computational modeling for prospective design as well as rapid manufacture of such materials, products, devices, therapies, materials, and parts. This technology report lays out the process for the novel Microscopy Aided Design and ManufacturE (MADAME) technology platform (1), spanning ideation through to manufacture of such smart materials, products and parts. *Smart* in this context refers to materials that respond to stimuli in their environment, adapting their own structure to their prevailing environment, in short and/or long time periods with respect to design life, and thereby augment function through their entire lifecycle.

**Figure 1:**
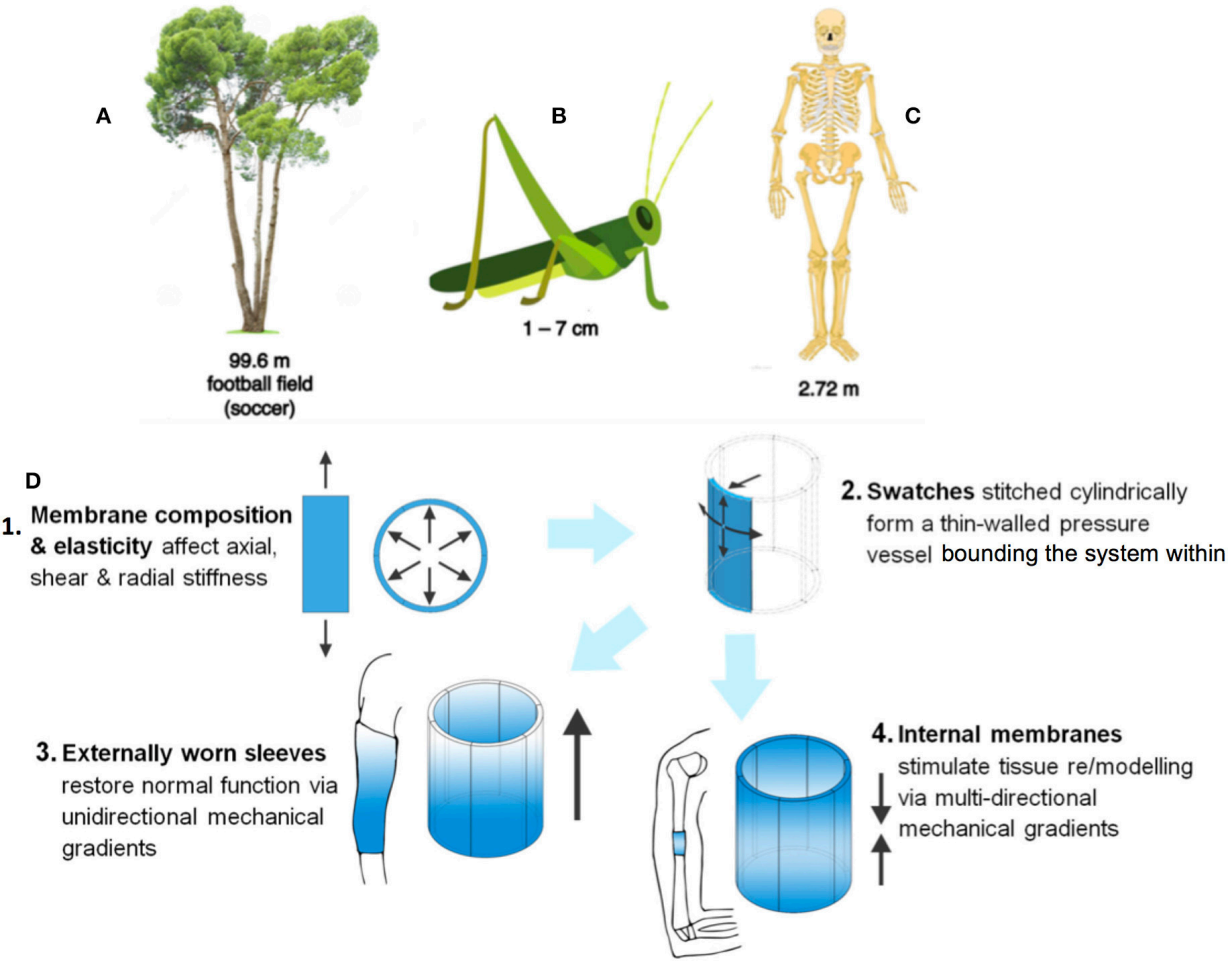
MADAME describes a design and manufacturing process that is applicable for the creation of diverse materials exhibiting unique gradients in mechanical structure. These gradients underpin the remarkable higher order function of such structures. For example, **(A)** the towering eucalyptus tree that bends like a blade of grass in high winds, **(B)** the mechanical gradients intrinsic to joint function in insect exoskeletons, and **(C)** the internal musculoskeletal system of vertebrates are all enabled through prescient distribution of mechanical properties in space and time. Nature provides infinite patterns that provide inspiration for ideation of smart materials. **(D)** Such mechanical gradient properties can be implemented to harness natural movements (**D1, D2**) for external (wearables, **D3**) and internal (implants, **D4**) applications that harness the movement of the local system e.g., to deliver directional pressure gradients and/or gradients in strain at interfaces. Figure adapted and used with permission (2).

The impetus for MADAME's development lies in the acute need to engineer and manufacture materials, products, and devices that emulate the smart mechanical and transport properties of nature's own (Figure 1). Nature abounds with advanced, stimuli responsive materials, that if emulated, provide new solutions to currently untenable design problems. Such problems include the discrepancy between the human life span and the design life of the human hip and its contemporary implant replacement. Human joints offer complex geometrical solutions to increase range of motion and stability during daily activities, e.g., ball and socket for the hip or complex composite bone and composite bone and ligamentous structure of the plane synovial acromioclaviular joint. Yet, novel design solutions may emulate emergent properties of natural joints and springs. For example, the eucalyptus tree exhibits a gradient in mechanical properties, enabling it to bend like a blade of grass under gale force winds while transporting nutrients upwards of 100 meters from the roots to the tip. At a different length scale, the grasshopper knee also exhibits gradients enabling “jointedness” and an intrinsic leaf spring. While 3D printing offers advantages with regard to rapid manufacturing materials and parts with mechanical gradients, it shows distinct disadvantages in particular for parts exposed to bending and tension (1, 3, 4). Recent advances in 4D printing incorporate actuator and sensor functions intrinsic to i.a. piezoelectric properties of 3D printed pieces (5–9), engineering of residual stresses into parts that can transform their geometry reversibly via folding (10–12). One such disruptive 4D printing modality harnesses natural movements, e.g., of the wearer or attributable to nature's cycles (tidal, weather, seasons, etc.), to design novel wearables and smart systems. MADAME uses computer-aided additive manufacturing incorporating three dimensional (4D) printing and computer-controlled weaving to create composite design motifs that emulate tissue patterns of woven protein fibers (3, 4), gradients in different caliber porosities, and mechanical and molecular properties intrinsic to tissues (13, 14). In so doing, MADAME enables a new genre of smart materials, products and replacement body parts that exhibit advantageous properties in bending and tension as well as in compression and materials that harness forces linked to physiological activity to activate material properties.

This Technical Report introduces the MADAME technology platform and the technical background of the new technology and its applications. Advantages and disadvantages of the approach are discussed in context of future directions.

### Recursive Logic and Weaving of Textiles With Biophysical and Spatiotemporal Patterns

**Figure 2:**
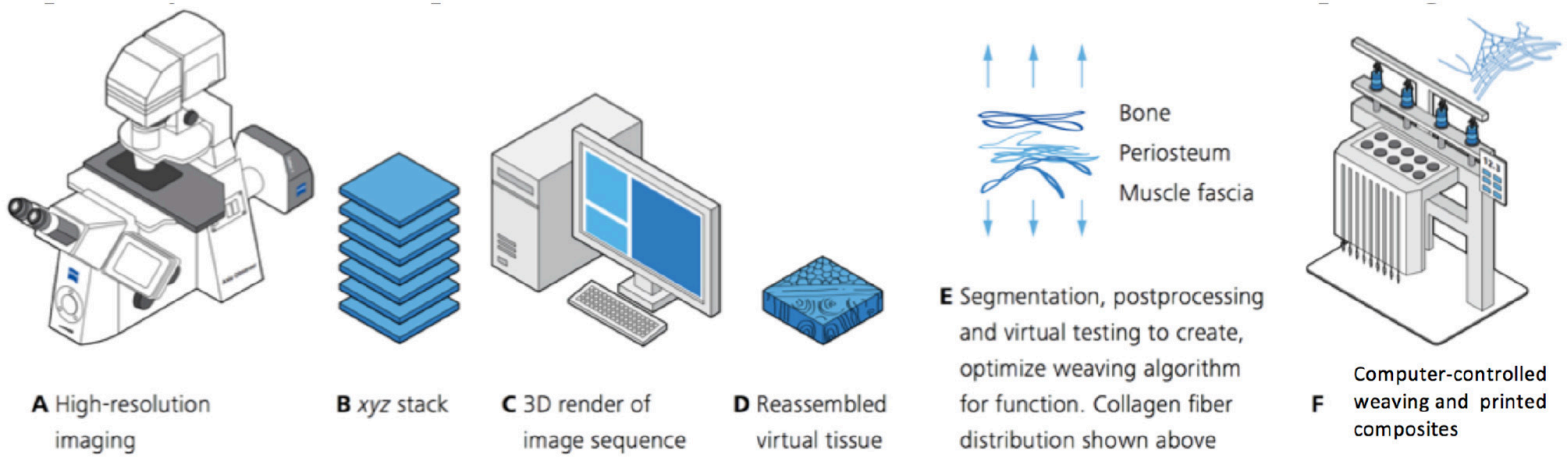
”Pipeline” for microscopy-enabled, scaled-up computer-aided design, and manufacture of composite multifunctional textiles and 3D prints emulating the body's own tissues, on hand from an example mapping, and weaving patterns emulating those of structural proteins, collagen, and elastin, in tissues. “Pipeline” describes the process of acquisition, filtering, and transformation of data, taking the raw data as input, processing it, and producing a final result as the end process in the pipeline. **(A–D)** Second harmonic generation and two photon microscopy of tissues reveals a spatial map of elastin and collagen, e.g., in the periosteum, a soft, and elastic tissue sheath that bounds all non-articular surfaces of bone. In this example, microscopy is used to map the precise pattern of elastin and collagen in native tissue. As the initial step in the pipeline, the raw microscopy data is thus transformed to patterns of representing material properties, e.g., stiffness. **(E)** These tissue maps are then rendered using computer-aided design software, where the patterns can be optimized for desired design specifications. This step in the pipeline creates stl files that are input into rapid manufacturing processes including e.g., integrated weaving and/or multi-dimensional printing. **(F)** Optimized designs thus provide inputs for computer controlled weaving of textiles and combined printing of composites that emulate the tissue studied under the microscope. This end process in the pipeline results in novel composite textiles that can be implemented in multiple fields of use. Figure adapted from (1) and used with permission.

MADAME describes the novel process of mapping spatial and temporal properties intrinsic to nature's smart materials, using imaging, and advanced computational methods (Figures 1, 2) (1, 3). The patterns intrinsic to such materials are then recreated using recursive logic. Remarkably, the loom was the earliest computer–prior to the first punch card driven computers, the Jacquard loom wove patterns using loops of paper with holes to guide when hooks fell through the paper loop (hook down) or stayed above the loop (hook up), thereby encoding binary patterns of e.g., tapestry weaves (1, 3). Recursive logic provides a basis for computer coding algorithms (15) and computer-controlled Jacquard looms enable creation of physical embodiments (textiles) of mechanical and other biophysical and spatiotemporal patterns intrinsically encoded in natural materials.

**Figure 3:**
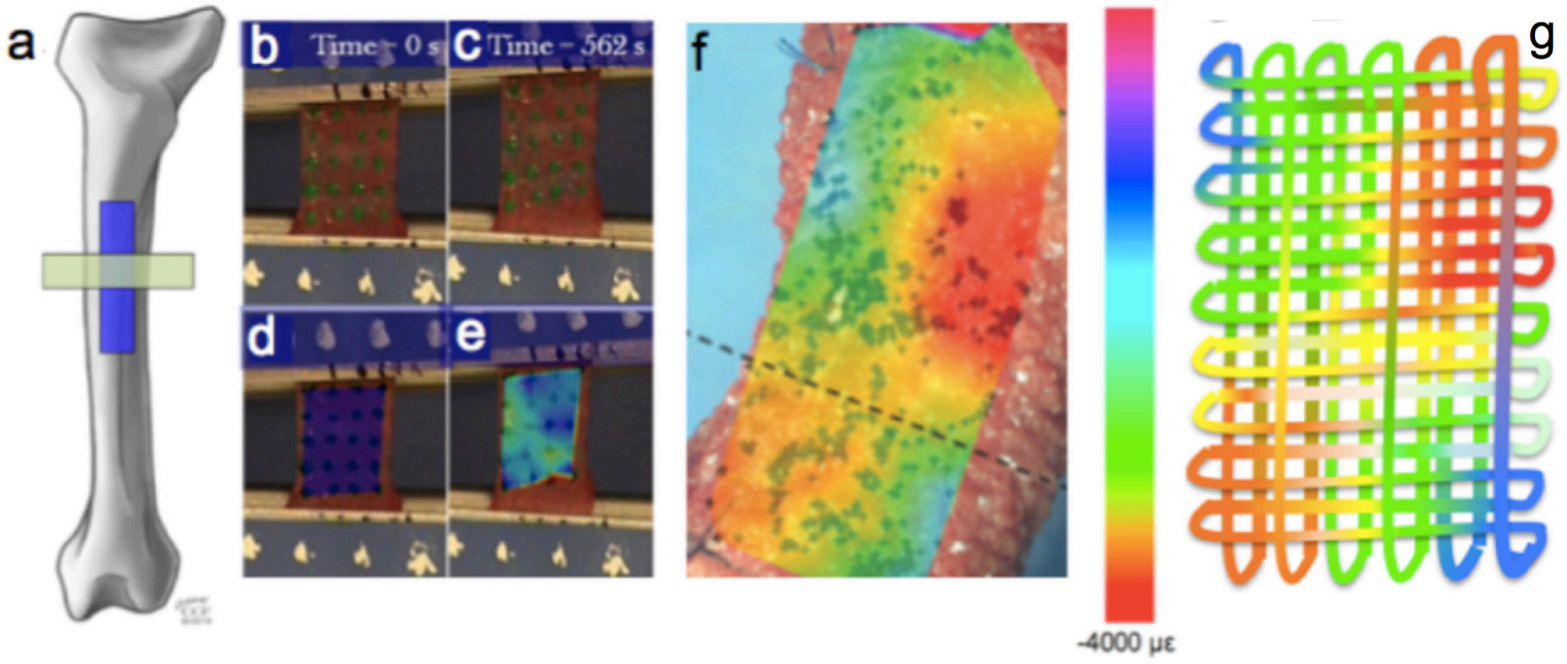
Recursive weaving of advanced materials that emulate Nature's own. **(a–e)** Example depicting anisotropic mechanical properties of periosteum, the hyperelastic sheath covering all bony surfaces in vertebrates. In the sheep femur **(a)** strain maps are created during loading in tension using digital image correlation, on sections of periosteum **(a–e)** cut in either the longitudinal or circumferential direction **(a)**. High resolution strain maps of the entire periosteum of the femur, *in situ* during stance shift loading, show heterogeneity of mechanical properties in space and time over the course of the loading cycle [**(f)**, still image taken from single frame of digital video over the loading cycle]. **(g)** Conceptually, a singular solution to recursively weave the tissue fabric of the periosteum tested would be to “unravel” a single strand's mechanical properties that would vary along the entire length of the strand. Many more solutions exist through creation of fiber patterns comprised of elastic and tough fibers such as elastin and collagen using computer-controlled weaving (1, 3, 4). Figure adapted from (1, 16–18) and used with permission.

The MADAME technology was developed to emulate the intrinsic weaves of natural tissues, from tree bark to grasshopper joints to human skin and bones. As an example, the patterns of structural proteins including elastin and collagen which imbue tissues with their respective elastic and toughness properties can be recursively mapped out and then imported into computer aided design files to weave textiles with scaled up mechanical property patterns mimicking those of the natural tissue (Figure 3) (1, 3, 4). In this way, the Jacquard loom technology provides a platform to create patterns of a variety of biophysical properties instead of its traditional use for the creation of color patterns in fabric and/or tapestries. Modern computer-controlled looms provide a rapid manufacturing method enabling control over 5,000 individual fibers, which themselves have different physical properties such as elasticity, respectively, stiffness. Composite materials are thus created in combination with 3D printing.

### Mapping of Hierarchical Porosities in Natural Tissues

A critical aspect of MADAME is the quantification and visualization of several orders of magnitude different length scale features within the same natural sample, which is often studied in the form of a histological section. The process from which patterns are derived from biological samples can involve recursive logic, as previously described, or clever image analysis approaches to identify and separate out (segment) different sized features, after which gradients can be described spatially, e.g., as heat maps, to better visualize their distribution in space and in relationship to each other.

In addition to the importance of mechanical property gradients in natural materials, porosity gradients provide transport pathways while also modulating mechanical properties of natural materials. As an exemplary case study, bone exhibits at least three levels of hierarchical porosity and gradients thereof (14, 19) which are characteristic to the tissue and which imbue the tissue with remarkable smart properties, such as counterintuitive flow properties (exuding fluid under compression and imbibing fluid under tension) (13, 14, 20–22), and flow directing transport areas of the tissue that are poorly vascularized, as well as providing direct conduits (resorption cavities created by osteoclasts) for osteoblasts to penetrate and lay down new bone in an oriented fashion, achieving anisotropic structural stability similar to reinforced concrete (21).

**Figure 4:**
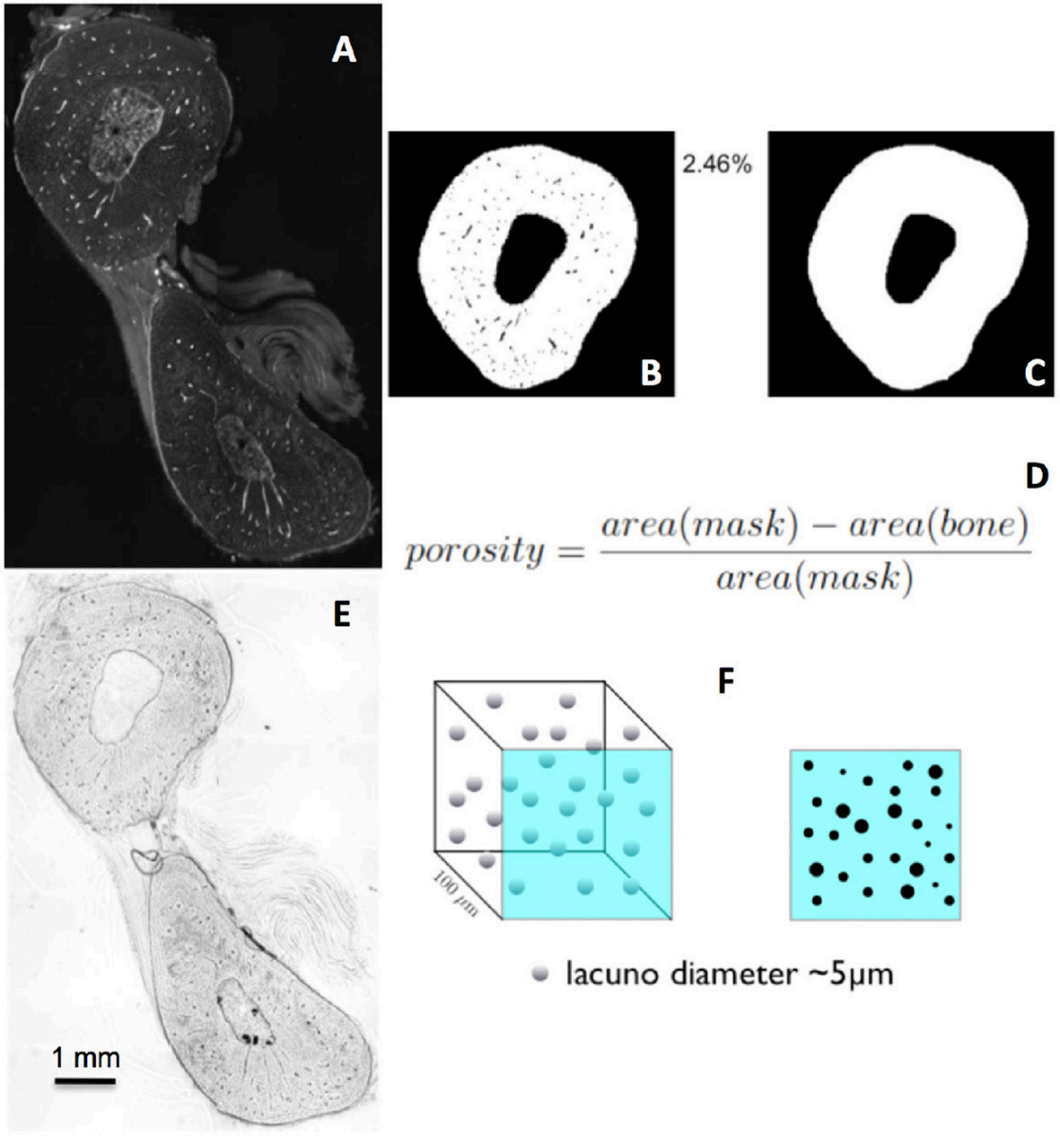
**(A–C)**. Mapping of the vascular porosity in bone. **(A)** Fluorescent confocal image. **(B)** Mask depicting area with vascular pores, *area*(*bone*) in the equation **(D)**. **(C)** Mask depicting area without vascular pores or *area*(*mask*) in the equation **(D)**. **(D)** Equation to calculate vascular porosity. **(E,F)** Calculation of lacunar porosity in bone, using **(E)** transmitted light images.

Automated segmentation and mapping of different calibers of porosity within the sample sample is a non-trivial problem. In the following case study we address the problem in detail for clarity and to allow for reduction to practice using different imaging modalities. To analyze porosity of whole bone cross-sections and multiple length scales, enabling spatial mapping and analysis of vascular porosity and pericellular porosity, a computer algorithm was developed in MATLAB (MathWorks, Inc., Natick, MA, United States) (14, 19). First, the vascular porosity of bone was mapped. High resolution confocal microscopy collages were acquired for the entire cross section of a histological sample containing a rat ulna and radius which had been injected intravitally with a 300 Da fluorescent tracer (Figure 4) (9). Vessels were identified automatically using the MATLAB algorithm and a mask of bone devoid of vessels was created to segment bone and calculate internal porosity. In this particular sample, the vascular porosity made up 2.46% of the cross sectional area of bone (Figure 4).

**Figure 5:**
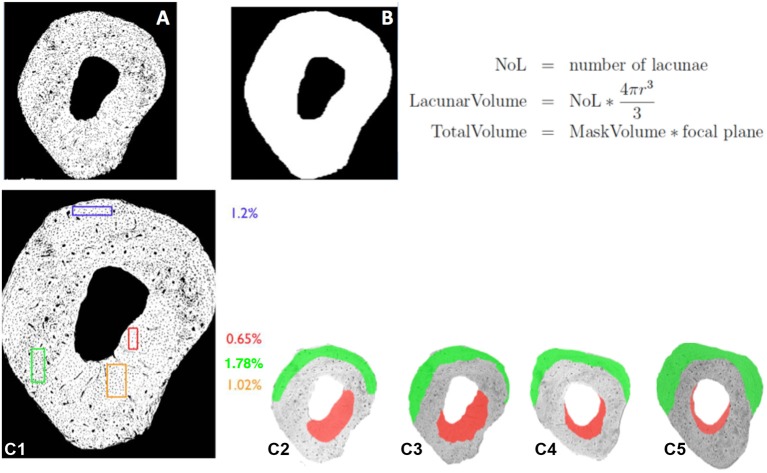
Mapping of the lacunar porosity in bone using transmitted light images **(A,B)** and mapping of site specific lacunar porosity in bone **(C1–5)**. **(A)** Mask of bone with lacunae. **(B)** MaskVolume of bone without lacunae. Based on the calculations, the lacunar porosity is 1.1% for the example shown. **(C1)** Different colors represent different lacunar porosities in specific sites of the cross section. **(C2-5)** Color plots depicting regions on different cross sections exhibiting characteristic porosities, e.g., **1.78** and **0.65%**.

To calculate the cell-length scale lacunar porosity (the lacunae are the voids in which the cells reside), transmitted light images were used similar to the way that the confocal images were used to calculate vascular porosity in the previous example. A mask was created, first without porosity, and then the lacunar porosity was calculated in 100 micron thick samples. The different caliber pores were identified as vessels and lacunae, while also accounting for the volume (Figures 4E,F). The lacunar porosity was calculated by generating a mask without porosity, and calculating the number of lacunae (Figures 5A,B), resulting in a lacunar porosity of 1.1% for the example. This process was then carried out for specific areas around the cross section to determine the site specific lacunar porosity (Figures 5C1–5).

**Figure 6:**
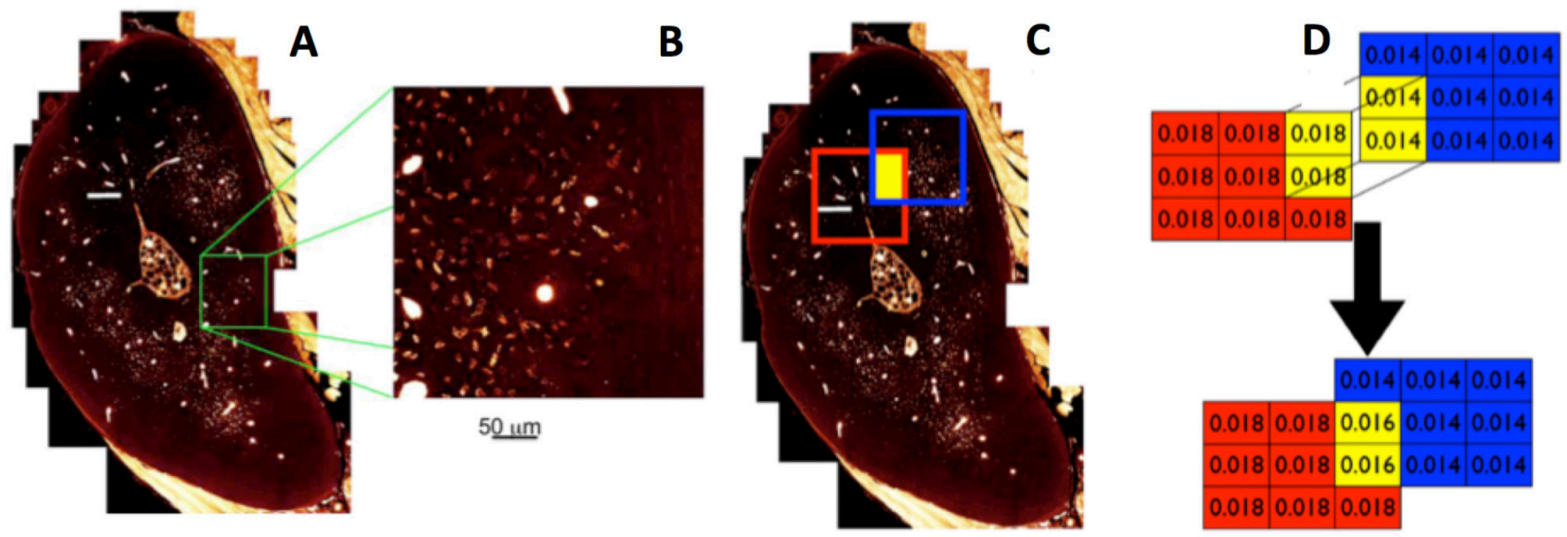
From high resolution maps of different caliber porosities [vascular, lacunar–**(A,B)**] to generation of matrices representing imaging data **(C,D)**.

**Figure 7:**
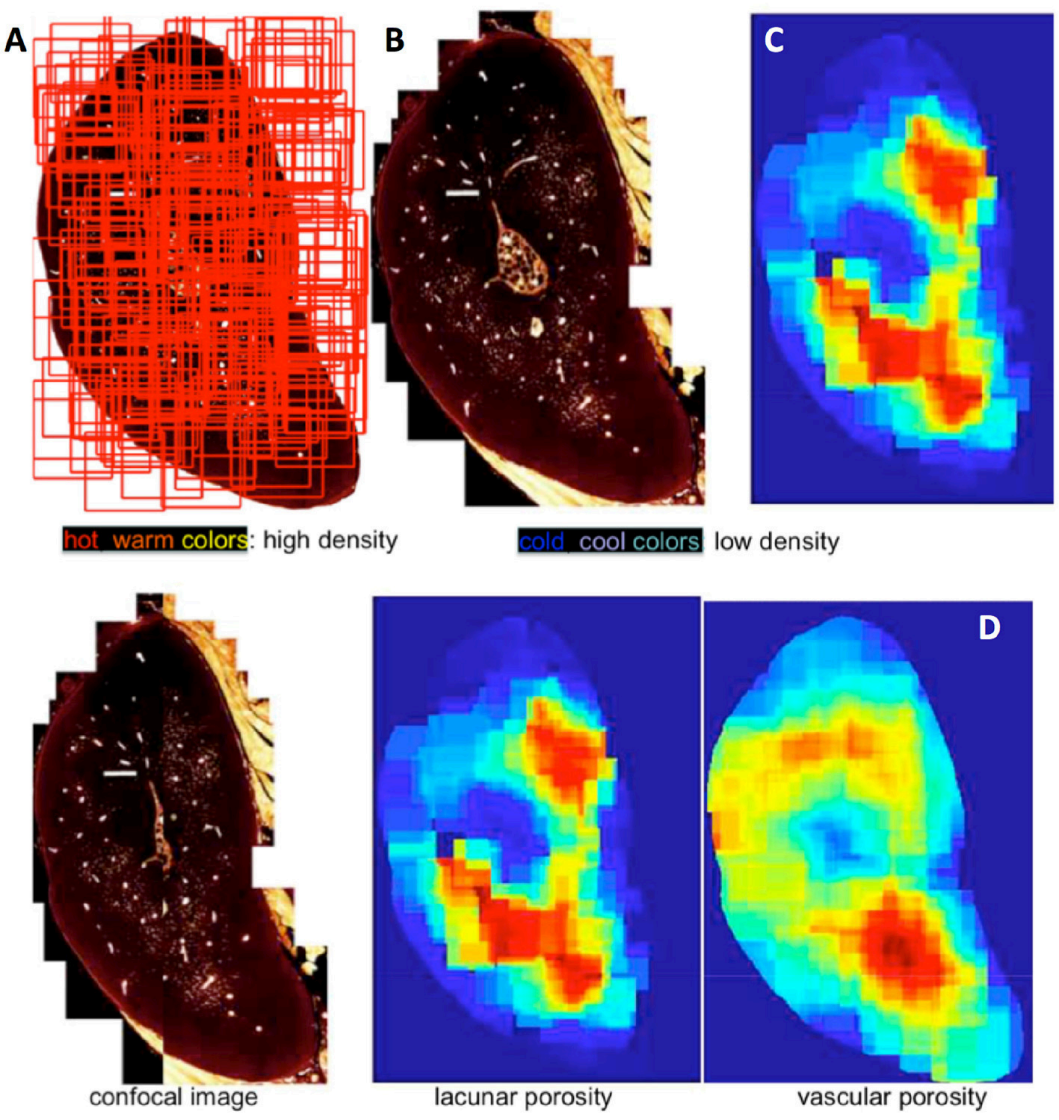
Heat maps are generated from random assessment of areas **(A)**, for lacunar and vascular porosity **(B)** in this case, and depicted as density gradients **(C,D)**, using hot-warm colors (red, orange, yellow) and low density using cool colors (blue, green). Images adapted from Knothe Tate et al. (14) and used with permission.

Then the site specific distribution of the vascular and lacunar porosities that make up the transport pathways were mapped using collages of high resolution confocal images (Figures 6A–C), which are depicted as “heatmaps” (warm colors represent high densities of particular features and cool colors reresent respective low densities, Figure 7). The logic underpinning the “heatmaps” forms the basis of a MatLab algorithm. In short, the measured porosity values are displayed in the form of color contour plots. These plots resemble the false color images obtained from imaging. MATLAB stores most images as two-dimensional arrays (i.e., matrices), in which each element of the matrix corresponds to a single pixel in the displayed image. A matrix with exactly the same dimension as the input image comprises all zero values. Next a randomly chosen region in the image is analyzed and two outputs are calculated including number of lacunae per area and vascular pores per area. These two parameters are then linked to the region in a way that the values are assigned to every matrix element representing the randomly selected area. Repeating this procedure several times causes regions to overlap (Figure 6D). Overlapping regions are averaged (Figure 7A), which leads to a good representation of the output-data over the cross-section if enough iterations are performed. In this way, a heat map of density of pores of two different calibers is created for the entire cross section, with warm colors depicting areas of high density and cool colors depicting areas of low density of e.g., lacunar and vascular porosity (Figures 7C,D).

This algorithm can be used to co-register images and their collages from imaging modalities as diverse as confocal laser imaging (yielding e.g., porosity gradients) (22, 23), second harmonic imaging (yielding e.g., collagen and elastin fiber gradients) (4), atomic force and electron microscopy (24, 25), multibeam scanning electron microscopy (26), computed tomography, magnetic resonance imaging (27), etc. These data sets, when encoded in computer aided design and computer aided manufacture file formats, serve as inputs for combined weaving of fiber patterns and multidimensional advanced manufacture (e.g., 3D printing or laser sintering) of porous structures. This enables creation of composite materials with strength in tension and bending and with smart, poroelastic properties such as flow directing materials. Hence, MADAME can be used to create novel materials and parts with gradients in poroelastic properties emulating those found in smart, natural materials.

### Additive Manufacturing of Scaled Up Natural Properties, Including Pore Gradients

Encoded in computer aided design and computer aided manufacture file formats, i.a. stereolithography (stl) or 3D Manufacturing Format (3MF) files, spatial plots of features provide inputs for additive manufacturing of materials, products, and parts that exhibit gradients and/or distributions in properties of natural materials. Additive manufacturing can take place via either computer-controlled weaving and/or additive manufacturing processes including i.a. stereolithography, powder sintering, 3D printing, etc. and/or electrospinning, weaving, and knitting.

The order and/or combined processes of weaving, knitting and spinning with 3D printing can be tuned to achieve the desired final properties of the materials, products and parts. For example, a weave can be placed within a stereolithography bath, enabling polymerization of polymeric matrix in gradients defined by scaled microscopy data around the weave. Similarly, with laser sintering, apatite and other mineral or metal based powders can be sintered around the weave. Integrated weaving and 3D systems will enable the weaving of textiles within the monomer baths using jets instead of hook-based weaving looms that are completely integrated with 3D printing modalities (18).

**Figure 8:**
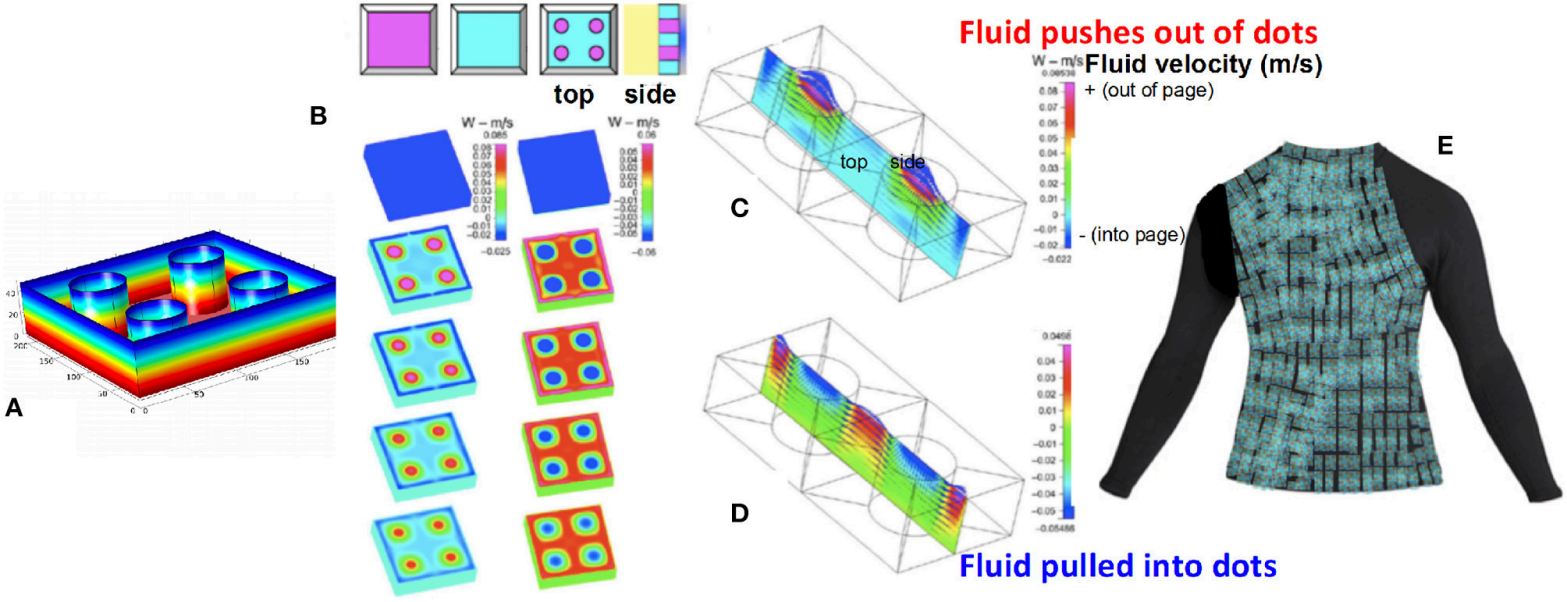
Application of MADAME to designer dressings and wearables. Modular designs **(A)** can be scaled up and tuned e.g., for bespoke bandages with spatial and temporal control of drug delivery. **(B–D)** Directionality of delivery dots and surrounding areas can be controlled by the architecture of the module. Scale bars depict fluid velocity, with warm colors indicating flow outwards and cool colors, flow inwards; e.g., pushing on the patch **(B,C)** results in flow out of the delivery dots. **(E)** Example of large scale, wearable wound dressing for e.g., burn treatment. Images recreated with permission from (14, 17).

Thus, we have described a pipeline or machine-based workflow (Figure 2) to design and manufacture smart dressings, drug deivery patches, and replacement body parts using MADAME. While “pipeline” refers to data driven processes that execute on the order of minutes and hours, “workflows” have more human interaction and periods of execution can be extend to days and years. MADAME shows great promise for the realization of new classes of materials, products and devices that will benefit patients, allowing for incorporation of unprecedented spatial and temporal patterns. One example is a new class of “designer” wound dressings *cum* delivery devices that are tuned to the spatial and temporal wound healing and drug release kinetics of individual patients, that harness the patient's movements to facilitate delivery, and that signal the wearer or the carer when the active ingredients are spent (Figure 8). This application can be further expanded as a disruptive platform for development of new classes of wearable materials and devices as well as internal applications, such as implants and medical devices.

**Figure 9:**
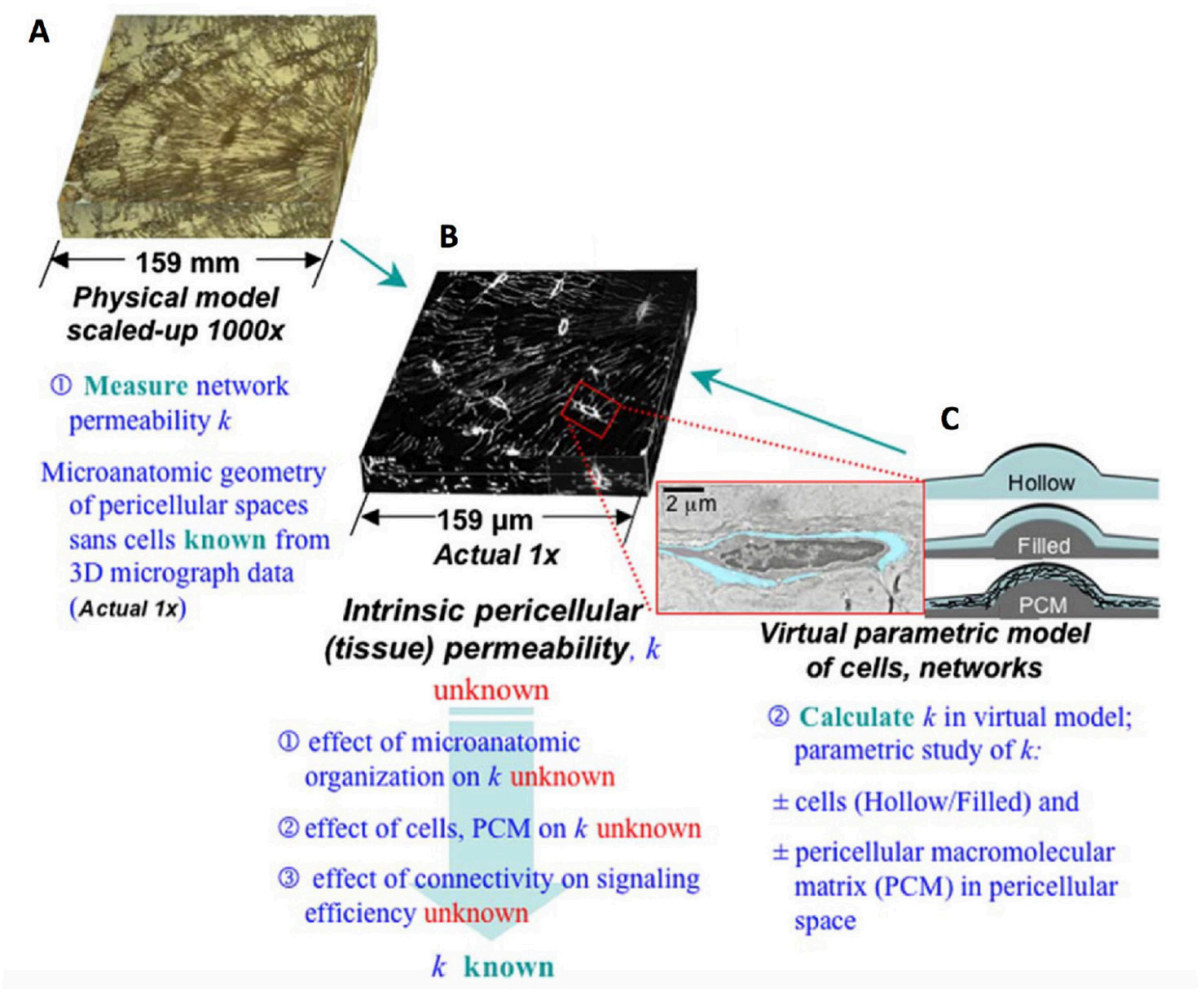
Early example of scale up and rapid prototyping of micron scale systems to emulate smart permeability properties in 1,000x scaled up (cm length scale) system. The intrinsic tissue permeability cannot be measured based on microscopy alone **(B)**. 1,000x scaled up physical renderings of the microscopic data are depicted as inverse microscopy data to encode flow around cells and their networks **(A)**. Virtual renderings of single cells enable analysis of the effect of pericellular matrix permeability on bulk pericellular tissue permeability **(C)**. Only through parallel study of virtual, scaled up physical renderings, and virtual *in silico* modeling based renderings of the system at different length scales, can the interactions between the elements and bulk properties of the tissue be estimated and validated. These studies were the first of their kind and they paved the way for organ to nano scale maps of human tissues and organs using other imaging modalities. Images used with permission after (28).

The pipeline has been tested on scaled up, three dimensional confocal microscopy datasets of the pericellular space in cortical bone (Figure 9). In this case, volumetric microscopy data was inverted to represent the fluorescent-dye infused cellular features as voids, and approximated in stl file format. The stl files contain no scale information, i.e., can be scaled up or down and used as inputs to create physical renderings at any desired scale and using any compatible rapid manufacturing modality. The physical renderings thus created, e.g., via 3D printing, enable unprecedented measurements using similitude theory, where measures at actual length scale are scaled up and down from the physical rendering. Similitude is a powerful, classical tool in mechanical engineering, applied by Da Vinci through to the modern day (14, 22). In the current example of the pericellular fluid space in cortical bone, for the first time pericellular tissue permeability could be measured on scaled up physical renderings of actual tissues. Pericellular permeability measures are of particular relevance for predicting of pharmaceutical delivery kinetics at local and global length scales.

Similarly, the pipeline was tested and validated in scaled up patterns of structural proteins mapped in ovine periosteum, an elastic and soft tissue sheath covering all bone surfaces and providing a niche for stem cells (1, 3, 4, 29–32). For the first time, using MADAME it was possible to create textiles that emulate the smart mechanical properties of the periosteum. The value proposition of MADAME is to scale up gradients in i.a. mechanical properties, porosities, and protein patterns to rapid prototype new materials that emulate patterns in natural materials. This provides an unprecedented means by which smart properties of natural tissues and systems can be mapped precisely using high resolution microscopy and used as a basis for manufacturing of scaled up materials that emulate nature systems.

**Figure 10:**
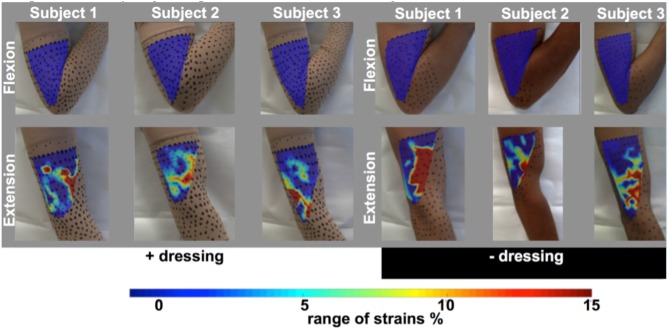
Coupled experimental mechanics and modeling studies enable determination of the range of strains on the surface of the human arm typical for daily activities. Digital imaging correlation methods and custom computer code developed for mapping strains *in situ* on the surface of the periosteum (*cf*. Figure 3) were used to measures strain on the surface of the arms of three subjects, with and without the presence of a compressive dressing. Strains are mapped at one point in time (one frame of digital video) during flexion and compression of the arm. Figure after (33) and used with permission.

The pipeline can be further tailored to best harness the wearer's natural movements and thereby to e.g., augment transport to and from the wound surface via material design that directs convective flow by harnessing displacements at the interface with the skin (Figures 8, 10). Thus, MADAME integrates inputs encoding material properties in context of the physiological mechanical environment in which the thus designed and manufactured products will be used, which provides independent and synergistic optimization of materials design and manufacture.

The inherent advantages and disadvantages of the enabling and disruptive MADAME technology align with those of current 3D- and 4D-printing technology platforms (Table 1). The major advantage of MADAME over current 3D- and 4D-printing modalities is that provides a means to manufacture novel composites with biophysical and spatiotemporal gradients and associated sensor and actuator functions that harness natural movements or transformations. The major disadvantages of MADAME include the need for high resolution imaging that crosses length scales, as well as cutting edge testing and validation, both of which requires operators with multidisciplinary, technical, and soft skillsets. With increasing sophistication of manufacturing and design capabilities, the need for creation of a future workforce with multivalent skillsets as well as creative ideation capacity will increase in importance, which in turn will drive the need for an educational curricula and training opportunities to gain those skillsets. At the same time, opportunities for integration of clean manufacturing methods and for employment of a highly educated workforce presents new opportunities for economic growth in geographical regions with lagging traditional manufacturing sectors.

**Table 1:** Comparison of the relative advantages and disadvantages of MADAME, 3D- and 4D-printing.

	**Advantages**	**Disadvantages**
3D printing	Off the shelf technology widely available and implementable	Limitations with regard to practical achievement of seamless, high resolution gradients in properties Lack of actuation and sensing in its simplest form Need for enabling technologies to prepare materials so they can be implemented using additive manufacturing processes such as stereolithography, laser sintering, etc. and limitation to combining materials in a single system
4D printing	Adds “smart” functionality to 3D printed materials, including actuation and sensing functions Disruptive platform - can be used to invent novel products and devices with wide range of applications in different industry sectors	Disadvantages of 3D printing may apply but may be overcome if functionality is added independent of 3D printing logistics, *e.g*. engineering in residual strains Implementation is initially specific to functionality added
MADAME	Can be implemented using 3D and 4D printing Enables creation of novel composites with biophysical and spatiotemporal gradients intrinsic to fibers and printing medium making up the materials Novel composites with biophysical stimuli activation depending on choice of fibers and surrounding matrix and their respective integration	Disadvantages of 3D and 4D printing may apply but may be overcome if functionality is added independent of 3D/4D printing logistics, e.g., using composite manufacturing methods Requires high resolution imaging platforms that enable cross scale imaging Requires state-of-the-art/cutting edge testing and validation Requires multidisciplinary skillsets

## Discussion and Conclusions

Using rapid prototyping, we expect the next generation of external and internal wearables including garments and implants, designer dressings, and drug delivery devices to be customizable and 3D printable in the General Practitioner's or nurse's office, and/or at the drug store/chemists. In the future, such devices will exhibit novel functionality, from delivery of drugs and biologics including stem cells, to active collection and monitoring of wound exudate, to modulation of the wound healing cascade through spatial and temporal presentation of factors that modulate cell behavior (migration, adhesion, proliferation, differentiation). Through clever implementation of e.g., click chemistry, they will signal the patient when it is time to return to the medical provider for follow up care (or, alternatively they will signal the wound care team when it is time for dressing change).

Aging is associated with impairment to healing and repair processes. Mobility decreases with increasing age and, in conjunction with incidence of diabetes or other comorbidities, the incidence and challenges intrinsic to treatment of chronic wounds increases. Concomitant to the increasing incidence of difficult to treat wounds, providing care to the ever-increasing aged population presents significant societal and economic challenges. Ultimately this approach will facilitate repair and healing processes that promote longevity through the described pipeline to rapid prototype bespoke external (wearables) and internal (implants, medical devices) wound dressings that deliver drugs and take up wound exudate.

MADAME is paradigm shifting and its significance can be demonstrated by the fact that it addresses an important problem as well as a critical barrier to progress in the field. Applied to medical products, materials and replacement parts, MADAME will provide caregivers a new means by which to treat wounds and physical impairments in a manner that is doubly efficient in that it will facilitate and thereby speed wound healing while also reducing the burden to caregivers.

## Ethics Statement

The digital image correlation study of strains on the surfaces of human subjects' arms was carried out in accordance with the recommendations and with approval of the UNSW human ethics committee, University of New South Wales, Sydney, Australia (HC 14077). All subjects gave written informed consent in accordance with the Declaration of Helsinki.

The studies quantifying and creating spatiotemporal maps of mechanical and porosity properties across cross sections in tissues of sheep and rats was carried out in accordance with the recommendations of the Animal Care and Use Committee of the Canton of Grisons Switzerland, who approved the protocols.

## Author Contributions

HS and MKT carried out the coupled imaging and computational modeling porosity analyses. JD carried out the coupled digital image correlation and computational analysis of surface based strains of the arm under flexion and extension, in collaboration with JN, DH, and MKT. MKT conceived of the collagen and elastin mapping studies and JN carried them out to create the first MADAME based textiles emulating periosteum. EA and MKT carried out experimental and computational modeling research and development of the flow directing foams technology platform. MKT conceived of the flow directing foam technology, the recursive weaving and the MADAME platforms. MKT wrote the manuscript, which was read and approved by all co-authors.

### Conflict of Interest Statement

Several patent applications have been issued and/or are pending in relation to the work described in this technical report. While these technologies have the potential to generate revenues for the inventors, they are still in the preclinical R&D phase of the translational cycle. The intent of the technical report is to disseminate findings among the R&D community and to share the novel approach to med tech ideation, development, and translation. The authors declare that the research was conducted in the absence of any commercial or financial relationships that could be construed as a potential conflict of interest.
